# Reviewers list for *Perception* and *i-Perception* for 2025

**DOI:** 10.1177/20416695251410605

**Published:** 2026-01-08

Adiarte, Sandra

Agostini, Tiziano

Aivar, M. Pilar

A-Izzeddin, Emily

Akbarinia, Arash

Allen, Harriet

Allison, Robert

Andrews, Tim

Angelucci, Alessandra

Apthorp, Deborah

Asher, Jordi M.

Ashida, Hiroshi

Attard-Johnson, Janice

Bach, Michael

Baker, Kristen

Balas, Benjamin

Ballardini, Giulia

Balta, Eirini

Barnhart, Anthony

Barratt, Daniel

Barrionuevo, Pablo

Battaglini, Luca

Becker, Casey

Benedetto, Alessandro

Bennetts, Rachel

Berti, Stefan

Bertolasi, Jessica

Billig, Alexander J.

Billock, Vincent

Böing, Sanne

Bossard, Martin

Boutet, Isabelle

Bouyer, Loren

Bovet, Jeanne

Brascamp, Jan

Brenner, Eli

Brooks, Kevin

Bruns, Patrick

Burke, Darren

Burr, David

Calbi, Marta

Campana, Gianluca

Campus, Claudio

Cao, Xiaohua

Caplovitz, Gideon

Carragher, Dan

Cassidy, Brittany

Castellotti, Serena

Cavanagh, Patrick

Cavdan, Müge

Chen, Hui

Chen, Jing

Chen, Yan-Ru

Chien, Sarina Hui-Lin

Chu, Qian

Ciorli, Tommaso

Civile, Ciro

Cleworth, Taylor

Collerton, Daniel

Contemori, Giulio

Costa, Marco

Cristino, Filipe

Crookes, Kate

Culling, John

Curwen, Caroline

Cuturi, Luigi F. F.

Dalmaso, Mario

Dassonville, Paul

Davis, Greg

De Sá Teixeira, Nuno

de Vries, Ingmar

DeGutis, Joe

Dekker, Tessa

Devue, Christel

Di Stefano, Nicola

DiBianca, Steve

Dijkstra, Nadine

Dobs, Katharina

Doerschner, Katja

Dorsi, Josh

Doumas, Mihalis

Durgin, Frank

Eayrs, Joshua

Edwards, Mark

Ehrsson, Henrik

Ekroll, Vebjørn

Eldem, Umut

Erickson, William

Faul, Franz

Fitousi, Daniel

Foecker, Julia

Forster, Bettina

Forster, Pierre-Pascal

Foulsham, Tom

Frascaroli, Jacopo

Friedenberg, Jay

Fu, Qiu-Fang

Fu, Shimin

Fuchs, Xaver

Fukui, Takao

Fysh, Matt

Ganel, Tzvi

Gao, Xiaoqing

Gao, Yi

Garza, Ray

Gegenfurtner, Karl

Gerbino, Walter

Getz, Laura

Glicksohn, Joseph

Goffaux, Valerie

Goktepe, Nedim

Goupil, Nicolas

Grasso, Paolo

Graves, Jackson

Greenspon, Charles

Greenwood, John

Griffin, Lewis

Grove, Philip

Grueter, Thomas

Guarischi, Marta

Gvozdenovic, Vasilije

Haile, Julia

Hallez, Quentin

Harrison, Róisín

Havard, Catriona

Hayashi, Masamichi

He, Qing

He, Sheng

Heffer, Naomi

Heller, Morton

Herzmann, Grit

Hessels, Roy

Hesslinger, Vera

Hibbard, Paul

Hidaka, Souta

Hine, Kyoko

Hirst, Rebecca

Hoetink, Alex

Hollett, Ross C.

Honekopp, Johannes

How, Martin

Hubbard, Timothy

Hübner, Ronald

Hughes, Anna

Hughes, Barry

Hung, Shao-Min

Huntley, Michelle

Hurlbert, Anya

Ichikawa, Makoto

Im, Hee Yeon

Israr, Ali

Ito, Hiroyuki

Jano, Sophie

Jin, Haiyang

Jonauskaite, Domicele

Jordan, Tim

Jordan, Timothy

Jouffrais, Christophe

Jurgensen, Frauke

Juttner, Martin

Kamatani, Miki

Karaaslan, Aslan

Kelly, David

Kennedy, John

Kitaoka, Akiyoshi

Kiyokawa, Hiroaki

Koenderink, Jan

Kopiske, Karl

Kuessner, Mats

Kuling, Irene

Kuraguchi, Kana

Lacey, Simon

Laeng, Bruno

Laetitia, Grabot

Lages, Martin

Lander, Karen

Lawrence, Rebecca

Lee, Jasmine

Lee-Masson, Haemy

Legge, Gordon

Leiros Costa, Thiago

Leloup, Frederic Bernard

Leonards, Ute

Lewis, Michael

Liang, Kun

Linnunsalo, Samuli

Linton, Paul

Longo, Matthew

Lovell, Paul

Lucia, Mariapia

Ma, Shu

Magnotti, John

Mak, Marloes

Mamassian, Pascal

Manassi, Mauro

Marin, Manuela

Marotta, Andrea

Marzouki, Yousri

Mather, George

Mathôt, Sebastiaan

McCann, John J

McManus, Chris

Meese, Timothy

Mello, Antonio

Merfeld, Daniel

Mestry, Natalie

Mesz, Bruno

Miyawaki, Yu

Moraes, Renato

Morea, Martina

Morimoto, Takuma

Moscicka, Albina

Motoki, Kosuke

Mruczek, Ryan

Musa, Marianna

Nabil, Shoaib

Nagai, Takehiro

Nardini, Marko

Newell, Fiona

Niehorster, Diederick C.

Nieuwenstein, Mark

Niimi, Ryosuke

Nittono, Hiroshi

Norman, J. Farley

Olkkonen, Maria

Olszanowski, Michal

Oomen, Danna

Otazu, Xavier

Paffen, Chris

Palma, Tomas A.

Palmer, Colin

Paramei, Galina

Parker, Andrew

Parovel, Giulia

Peissig, Jessie

Penacchio, Olivier

Peviani, Valeria

Pizzolante, Marta

Plaisier, Myrthe

Plank, Tina

Pome, Antonella

Poncet, Fanny

Poncet, Marlene

Poom, Leo

Proulx, Michael

Qian, Cheng

Quinlan, Philip

Ramon, Meike

Reader, Arran

Riberto, Martina

Ricci, Raffaella

Ritchie, Brendan

Ritchie, Kay

Robinson, Chris

Robinson, Joseph

Roccato, Marco

Rogers, Brian

Rohe, Tim

Rose, David

Rudd, Michael

Sandford, Adam

Sapir, Ayelet

Saryazdi, Raheleh

Sato, Katsunari

Saurels, Blake

Sawayama, Masataka

Sayim, Bilge

Scheller, Meike

Schlüter, Nick

Schmidt, Timo

Schmidtmann, Gunnar

Schütz, Alexander

Sekuler, Robert

Shapley, Robert

Sharma, Saloni

Shenyan, Oris

Simmons, David

Singh, Tarini

Skarratt, Paul

Sokolov, Alexander

Soranzo, Alessandro

Spehar, Branka

Steward, Ben

Stojilović, Ivan

Storrs, Katherine

Stuit, Sjoerd

Sun, Zekun

Super, Hans

Szubielska, Magdalena

Takahashi, Kohske

Tammurello, Carolina

Tamura, Hideki

Tang, Xiaoyu

Taubert, Jessica

Teramoto, Wataru

Thornton, Ian

Todd, James

Tonelli, Alessia

Toscani, Matteo

Troje, Nikolaus F.

Turati, Chiara

Valarmathi, A.

Valsecchi, Matteo

van Dam, Loes

van der Smagt, Maarten

van Ee, Raymond

Van Lier, Rob

Velasco, Carlos

Verstraten, Frans

Vickery, Tim

Vitali, Helene

Voudouris, Dimitris

Vroomen, Jean

Wade, Nicholas

Wang, Lihui

Wang, Zhe

Ward, Nathan

Watson, David

Watt, Simon

White, David

Whitwell, Robert

Wiese, Holger

Wijntjes, Maarten

Wiley, Robert

Wincza, Radek

Witzel, Christoph

Woods, Andrew

Wyche, Nick

Xiao, Naiqi

Xie, Xin-Yu

Xiong, Ying-Zi

Yanaka, Kazuhisa

Yazdanbakhsh, Arash

Yildirim-Keles, Fazilet Zeynep

Yu, Linda

Zavagno, Daniele

Zhang, Huihui

Zhang, Jun-Yun

Zhao, Ke

Zhao, Yajun

Zhou, Guomei

Ziat, Mounia

